# FBXW7 expression is associated with prognosis and chemotherapeutic outcome in Chinese patients with gastric adenocarcinoma

**DOI:** 10.1186/s12876-017-0616-7

**Published:** 2017-05-02

**Authors:** Mao-Ran Li, Chun-Chao Zhu, Tian-Long Ling, Ye-Qian Zhang, Jia Xu, En-Hao Zhao, Gang Zhao

**Affiliations:** Department of Gastrointestinal Surgery, RenJi Hospital, School of Medicine, Shanghai Jiao Tong University, 160 Pujian Road, Shanghai, 200127 People’s Republic of China

**Keywords:** FBXW7, Gastric cancer, Prognosis, Chemotherapeutic outcome

## Abstract

**Background:**

FBXW7, a component of the Skp-Cullin1-F-box, mediates target protein recognition. It is a tumor suppressor gene that plays a role in the regulation of cell cycle exit and reentry via c-Myc, c-Jun and Notch degradation. There are few studies, particularly involving a large patient cohort, that have evaluated FBXW7 during gastric cancer progression.

**Methods:**

Our study aimed to evaluate the value of FBXW7 as a clinical marker in gastric adenocarcinoma (GC) patients including a subset treated with postoperative chemotherapy. Quantitative reverse transcription PCR (qRT-PCR) assay was used to measure FBXW7 transcript levels in tumors paired with normal gastric tissue in 24 gastric adenocarcinoma patients. Subsequently, 546 additional GC samples were evaluated from patients that underwent radical gastrectomy, including 118 early stage cases(Stage I) and 428 advanced stage cases (Stages II or III). Amongst the advanced stage patient cases evaluated, 347 received postoperative adjuvant chemotherapy. All 546 gastric adenocarcinoma cases were then evaluated by tissue microarray and immunohistochemistry (IHC) for FBXW7 expression. Clinicopathological features and diagnoses were confirmed by histopathologic evaluation and review of clinical data. Overall survival (OS) was then evaluated in the 546 gastric cancer patients.

**Results:**

By immunohistologic evaluation, low expression of FBXW7 in primary gastric cancer significantly correlated with poor differentiation of tumor cells. Moreover, low FBXW7 expression was associated with worse survival as well as worse adjuvant chemotherapy response.

**Conclusion:**

Our findings suggest that FBXW7 may serve as an important predictor in chemotherapeutic responses.

## Background

Gastric cancer is one of the most common worldwide malignancies with an increasing worldwide incidence rate of new cases, currently standing at more than 100 million cases per year. Gastric cancer is more common in China [1, 2] with a morbidity and mortality rate twice that of the worldwide average. Chemotherapy plays a key role in the treatment plan of patients with advanced gastric cancer [3]; however, the occurrence of chemotherapy drug resistance, especially multidrug resistance (MDR),limits its effectiveness [4]. At present, the mechanism of MDR remains unclear.

F-box and WD repeat domain-containing7 (FBXW7), an important member of the F-box family located on chromosome 4q32, was found by Leland while studying the budding yeast cell division cycle [5]. There are three protein isoforms:FBXW7α,FBXW7β and FBXW7γ. FBXW7αis considered the most important subtype as it is widely distributed in the human body. FBXW7 serves as a subunit of the SCF (Skp-Cullin1-F-box) complex [6]. The SCF complex participates in the ubiquitin-proteasome pathway reaction, a three-level cascade degradation of misfolded proteins, and is implicated in tumorigenesis as protein accumulation is thought to play a key role in tumor formation [7]. FBXW7 is widely considered to be an effective tumor suppressor gene [8, 9]. It has been shown that down regulation of FBXW7 can promote tumor cell proliferation. In turn, loss of FBXW7 alters the tumor gene expression profile and leads to increased lung cancer development in a murine model. FBXW7 can target the degradation of MCL1,cyclin E,Notch,c-Jun and c-Myc, cancer genes related to the proliferation and regulation of gastric cancer cells [10, 11]. MCL1 is highly associated with taxol resistance in malignant tumors derived from epithelial cells [12]. At present, FBXW7 down-regulation is found in numerous human malignant tumors, including non-small cell lung cancer, T cells leukemia, bile duct carcinoma, pancreatic carcinoma and endometrial carcinoma [9, 13, 14]. Although some studies indicate that low FBXW7 expression is associated with tumor progression and drug resistance in many malignancies [15, 16], there is a paucity of studies examining FBXW7 in gastric cancer. Therefore, the aim of this study was to explore the relationship between FBXW7 expression and the clinicopathological characteristics as well as chemotherapeutic outcomes in gastric adenocarcinoma patients.

## Methods

### Patients

Twenty-four freshly collected gastric cancer specimens paired with adjacent normal gastric tissue were obtained from radical gastrectomy surgeries performed from January 2013 to August 2014. Resected cancer and paired noncancerous tissues were immediately frozen in liquid nitrogen, and maintained at -80 °C until RNA and DNA extraction for quantitative real-time PCR was performed.

For FBXW7 immunohistochemical evaluation, GC patient inclusion criteria were as follows: 1) apathologic diagnosis of gastric adenocarcinoma; 2) no radiotherapy, chemotherapy, nor other anti-cancer therapies prior to the surgery; 3) radical tumor resection; 4) availability of complete clinicopathological and clinical follow-up data. The final date of follow-up was 31 December 2014 for all cases examined.

A total of 546 paraffin-embedded tissue samples met the previous criteria and were collected from GC patients at Department of Gastrointestinal Surgery, RenJi Hospital, Shanghai Jiao tong University, School of Medicine from January 2006 to December 2011 for tissue microarray construction and immunohistochemistry staining. Overall survival (OS) is calculated from the date of tumor resection until death.

A cohort of 347 patients underwent postoperative floxuridine-based chemotherapy treatment amongst the 546 GC patients examined. Tumor stage of all 347 patients was TNM stages II and stage III. Within this treated cohort, 84 patients were treated with a regimen including paclitaxel, 66 patients’ regimen included anthracycline, and 261 patients’ regimen included platinum. Dosage of chemotherapeutics per cycle were as follows: 1) docetaxel (Taxotere, Sanofi-Aventis, France) 75 mg/m2, intravenous; 2) oxaliplatin (Exolatin, Laboratoires Thissen, Belgium) 130 mg/m2, intravenous; or cisplatin 60 mg/m2, intravenous; 3) epirubicin (Pharmorubicin, Pfizer, USA) 100 mg/m2, intravenous; 4) fluorouracil 30 mg/kg, maintain 72 h by venous pump D2-D4. All patients with radical operation received adjuvant chemotherapy for 6 cycles for a period of 21 days.

### Total RNA extraction and quantitative real-time PCR

Total RNA was extracted from 24 fresh cancer tissue samples and paired normal tissue using Trizol reagent (Takara, Dalian, China) per the manufacturer’s instructions. The reverse-transcription reactions were carried out with random primers and M-MLV Reverse Transcriptase (Takara, Dalian, China) and resulting cDNA used for quantitative real-time PCR reaction via SYBR-Green method. All qPCR reactions were performed on a StepOneTM real-time PCR System (Applied Biosystems, Foster City, CA, USA). The specific primer sequences of FBWX7 and 18 s (18svedberg) were as follow: FBXW7 sense primer 5’-AAAGAGTTGTTAGCGGTTCTCG-3’ and antisense primer 5’-CCACATGGATACCATCAAACTG-3’. 18 s sense primer 5’-TGCGAGTACTCAACACCAACA-3’ and antisense primer 5’-GCATATCTTCGGCCCACA-3’. 18 s was used as an internal control. The 2-ΔCt method was used to quantify the relative FBXW7 expression levels.

### Tissue microarrays construction

TMAs were constructed by Shanghai Zhuo Li Biotechnology Co., Ltd (Zhuo Li Biotechnology Co, Shanghai, China). Because gastric cancer tissue is dispersed within gastric interstitial space, GC sample staining is necessary. Consequently, tissue paraffin blocks of GC samples were stained with hematoxylin-eosin to confirm the diagnoses and marked at fixed points with most typical histological characteristics under a microscope. Two 1.5 mm cores per donor block were transferred into a recipient block tissue microarrayer, and each dot array contained fewer than one hundred and sixty dots. Three-micron-thick sections were cut from the recipient block and transferred onto glass slides using an adhesive tape transfer system for ultraviolet cross linkage.

### Immunohistochemistry

The tissue microarray glass slides were baked at 55 °C for 1 h, and then de-paraffinized gradually through xylene, 50% xylene, and gradient concentrations of ethanol until immersed in tap water. Tissue sections were blocked for peroxidase activity with 0.3% Hydrogen peroxide at 37 °C for 30mins. Antigen retrieval was carried out via boiling in 10 mmol/L citrate buffer (pH 6.0) for fifteen mins. Then the tissues were incubated with FBXW7 antibody (diluted 1:100;mouse monoclonal anti-FBXW7 antibody [3D1] ab128062, Abcam, USA) overnight at 4 °C. Next day, the tissues were washed with phosphate buffer solution (PBS) for three times and incubated with goat anti-rabbit IgG-HRP (HUABIO) or goat anti-mouse IgG-HRP (ABCAM) secondary antibody for 1 h at room temperature. Immunostaining was carried out using diaminobenzidine substrate chromogen (Dako, Carpinteria, CA, USA) method and chromogenic reaction was controlled under microscope. After immunostaining, tissues were immersed into hematoxylin for nuclear staining. The slides were then dehydrated through gradient concentrations of ethanol, cleared with xylene, and cover slipped with neutral balsam.

TMAs were scored as follows: absence of any staining as score 0; weak-to-strong complete nuclear staining in <25% of tumor cells as score 1; weak-to-strong complete nuclear staining in 25–50% of tumor cells as score 2; strong complete nuclear staining in ≥50% of tumor cells as score 3. Score 0–1 was considered as low expression and a score of 2–3 was high expression for subsequent statistical analyses. All scoring was performed by 2 independent pathologists, who were blinded to clinical outcomes.

### Statistical analysis

Statistical analyses were conducted using SPSS 20.0 (SPSS Inc, Chicago, IL, USA) and GraphPad Prism 5 (San Diego, CA) software. FBXW7 mRNA in the cancerous and noncancerous tissues were compared using a paired-samples *t* test. The chi-square test was used to analyzed the relationship between FBXW7 expression and clinicopathological characteristics. OS curves were calculated according to Kaplan-Meier method and log-rank test was used for comparing the survival distributions. Univariate and multivariate analyses were based on the cox proportional hazards regression model. All statistical tests were two-sided. *P* value less than 0.05 was considered statistically significant.

## Results

### FBXW7 mRNA expression in 24 paired gastric adenocarcinomas and noncancerous gastric tissues

**Fig. 1:**
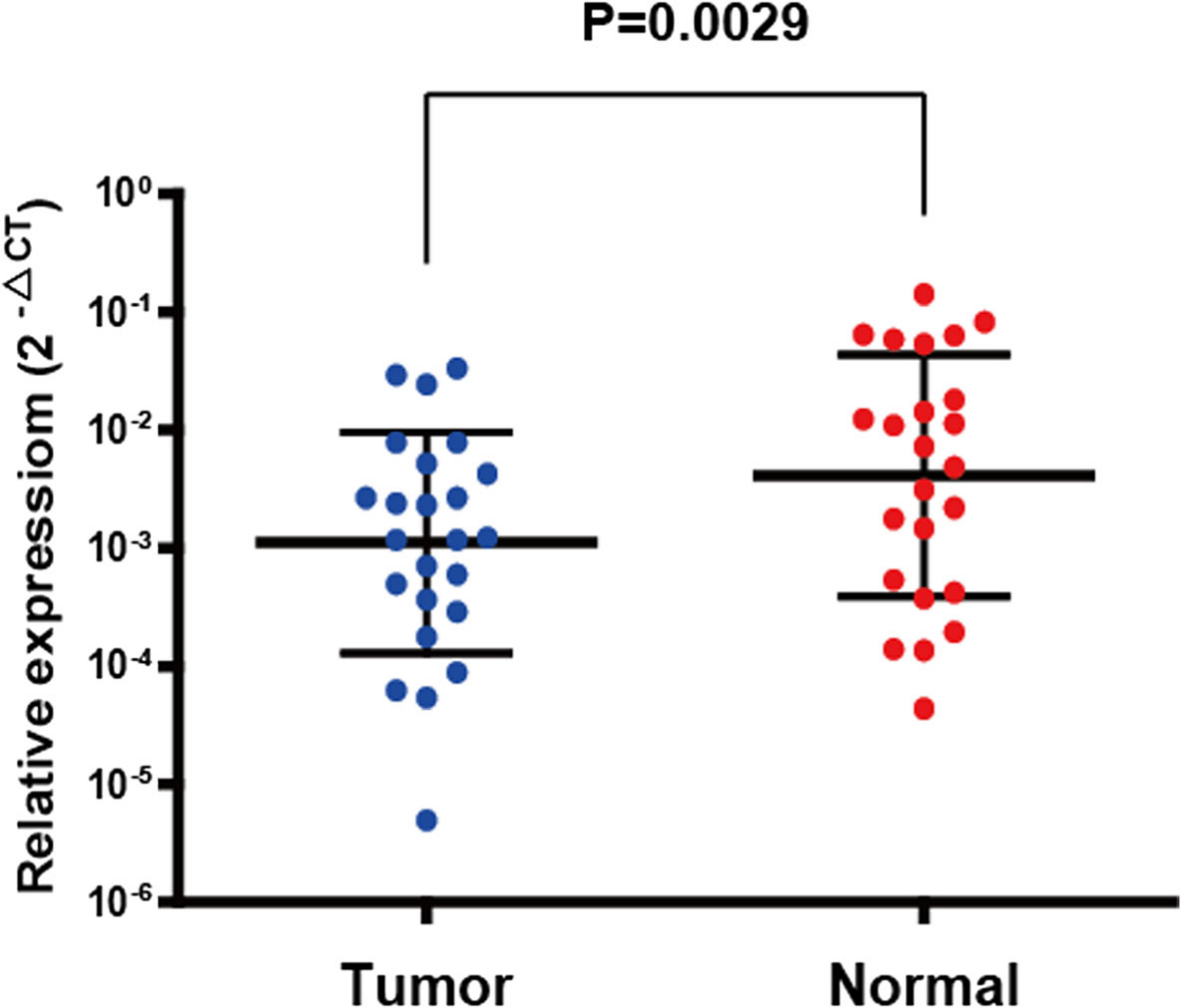
FBXW7 mRNA expression is decreased in paired cancerous and noncancerous tissue in 24 gastric adenocarcinoma patients. (*P* = 0.0029)

FBXW7 mRNA expression levels in gastric adenocarcinomas and paired noncancerous gastric tissue in 24 patients were examined by quantitative real time-PCR. 18 s was used as an internal control. A scatter dot plot for mRNA relative expression levels is provided in Fig. 1, and shows generally low transcript levels of FBXW7 in the adenocarcinomas compared to control tissue (*P* = 0.0029).

**Fig. 2:**
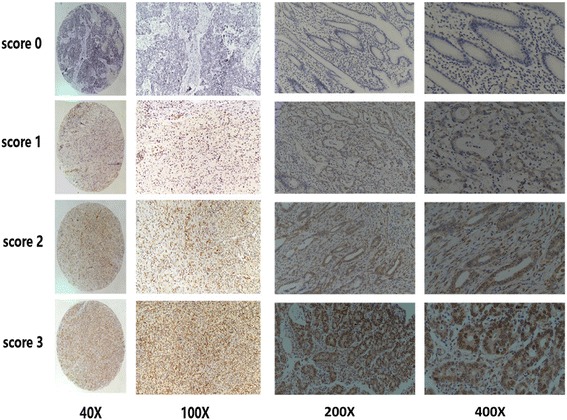
FBXW7 protein expression in gastric adenocarcinoma tissue sample: immunohistochemical representative images of FBXW7 expression scoring as 0, 1, 2 and 3. Images of 40X,100X, 200X AND 400X were showed in Fig. 2

*Correlation of FBXW7 expression with clinicopathological features in 546 gastric adenocarcinomas.* To confirm FBXW7 expression, we performed an immunohistochemistry (IHC) study evaluating all 546 GC samples. Representative immunostains of FBXW7 scored as 0, 1, 2 and 3 are shown in Fig. 2.

**Fig. 3:**
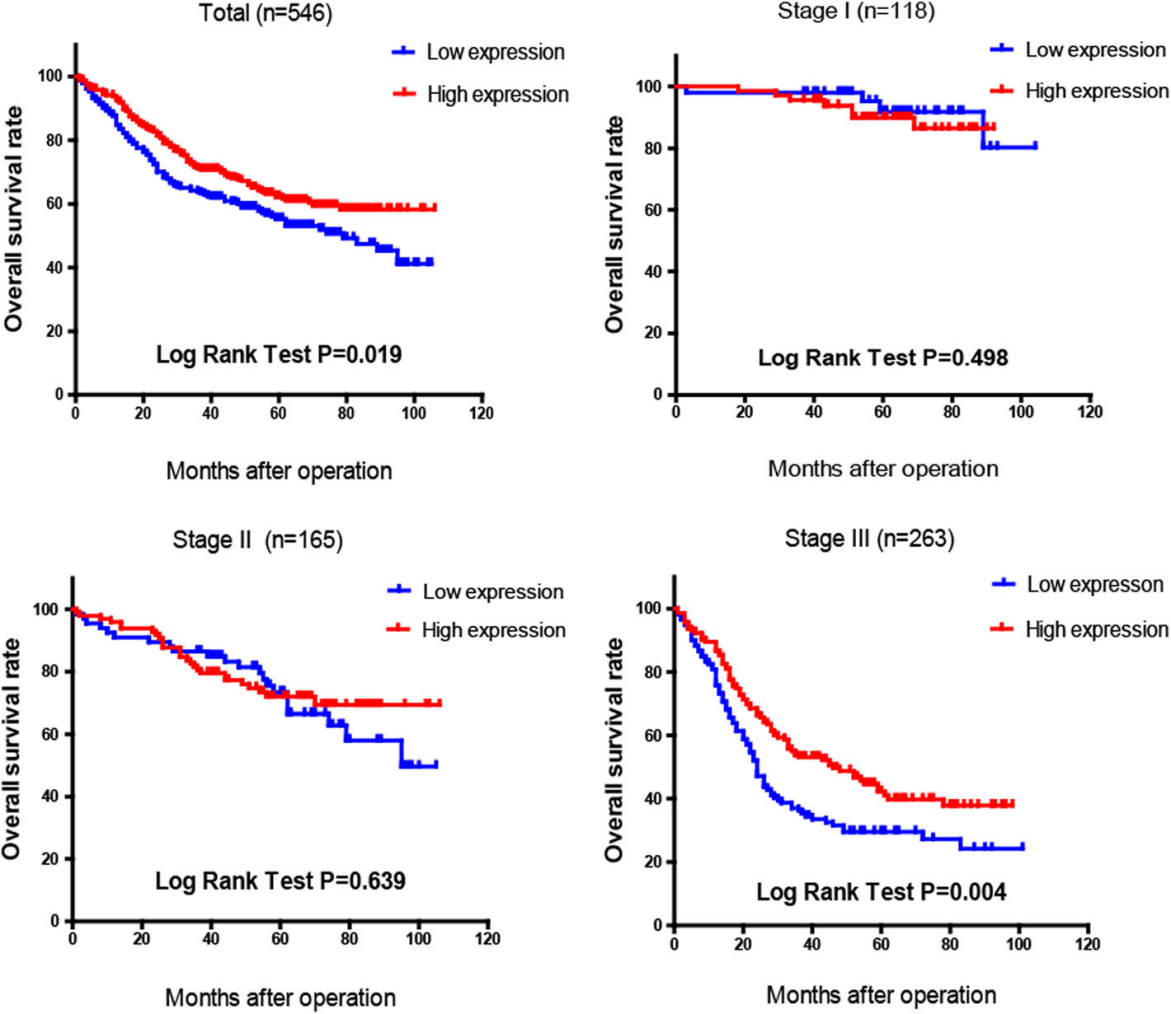
The overall survival curve of 546 GC patients demonstrated Low FBXW7 expression leads to poorer prognosis (*P* = 0.019). OS of stage III patients shows a significant difference between high and low FBXW7 expression groups (*P* = 0.004).OS of patients of stage I and stage II did not show a difference between the two groups

Correlation of FBXW7 expression with clinicopathological features was analyzed and is shown in Table 1. Tumor size (*P* = 0.001), differentiation (*P* = 0.001), nodal stage (*p* = 0.040) and vessel invasion (*P* = 0.003) were found to be significantly different between high and low FBXW7 expression (*P* = 0.001). However, no significant differences were found regarding age, gender, depth of tumor and nerve invasion. FBXW7 expression was significantly associated with prognosis in our gastric cancer cohort. The overall survival curve of 546 GC patients is shown in Fig. 3, which demonstrates low FBXW7 expression correlates with poorer prognosis. Furthermore, a significant difference was found between high and low FBXW7 expression groups and corresponding OS in Stage III patients (*n* = 263). This difference was not seen in Stage I or II patients.

**Table 1:** Correlation of FBXW7expression and clinicopathological characteristics in 546 gastric adenocarcinoma patients

	Number	FBWX7 low expression	FBWX7 high expression	*P*-Value
Age				0.182
≤ 60	253	118 (46.6%)	135 (53.4%)	
> 60	293	120 (41.0%)	173 (59.0%)	
Gender				0.473
Male	351	149 (42.5%)	202 (57.5%)	
Female	195	89 (45.6%)	106 (54.4%)	
Tumor size				0.001*
≤ 5 cm	348	133 (38.2%)	215 (61.8%)	
> 5 cm	198	105 (53.0%)	93 (47.0%)	
Histological grade				0.001*
Well	16	5 (31.3%)	11 (68.7%)	
Moderate	140	44 (31.4%)	96 (68.6%)	
Poor	390	189 (48.5%)	201 (51.5%)	
Depth of tumor (pT)				
T1	85	37 (43.5%)	48 (56.5%)	0.256
T2	76	27 (35.5%)	49 (64.5%)	
T3	149	61 (40.9%)	88 (59.1%)	
T4	236	113 (47.9%)	123 (52.1%)	
Nodal stage (pN)				0.040*
N0	207	91 (44.0%)	116 (56.0%)	
N1	100	32 (32.0%)	68 (68.0%)	
N2	107	48 (44.9%)	59 (55.1%)	
N3	132	67 (50.8%)	65 (49.2%)	
Vessel invasion				0.003*
Negative	444	180 (40.5%)	264 (59.5%)	
Positive	102	58 (56.9%)	44 (43.1%)	
Nerve invasion				0.148
Negative	479	203 (42.4%)	276 (57.6%)	
Positive	67	35 (52.2%)	32 (47.8%)	

*Statistical significant (*P* < 0.05)

### Correlation between FBXW7 expression and chemotherapy outcome in advanced stage gastric adenocarcinoma

**Fig. 4:**
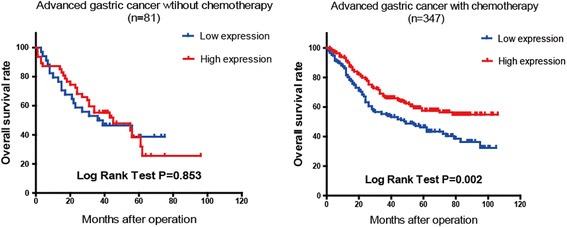
The overall survival curve of 347 advanced GC patients with postoperative chemotherapy show slow FBXW7 expression leads to worse chemotherapeutic outcome (*P* = 0.002). OS of 81 advanced patients without chemotherapy did not show a difference between the two groups

All 546 patients received a radical gastrectomy. Among which, 118 and 428 of the patients were diagnosed as early (Stage I) and advanced (Stage II or III) stages respectively. According to the 7^th^AJCC edition, these 428 advanced stage patients were classified into Stages II and III based on the post-operational pathologic diagnosis. Amongst the advanced stage cases, 81 patients declined chemotherapy. The OS curves of these advanced patients with or without chemotherapy are shown in Fig. 4. High FBXW7 expression was associated with improved survival for those who received chemotherapy (*P* = 0.002, Kaplan-Meier log rank test).

**Fig. 5:**
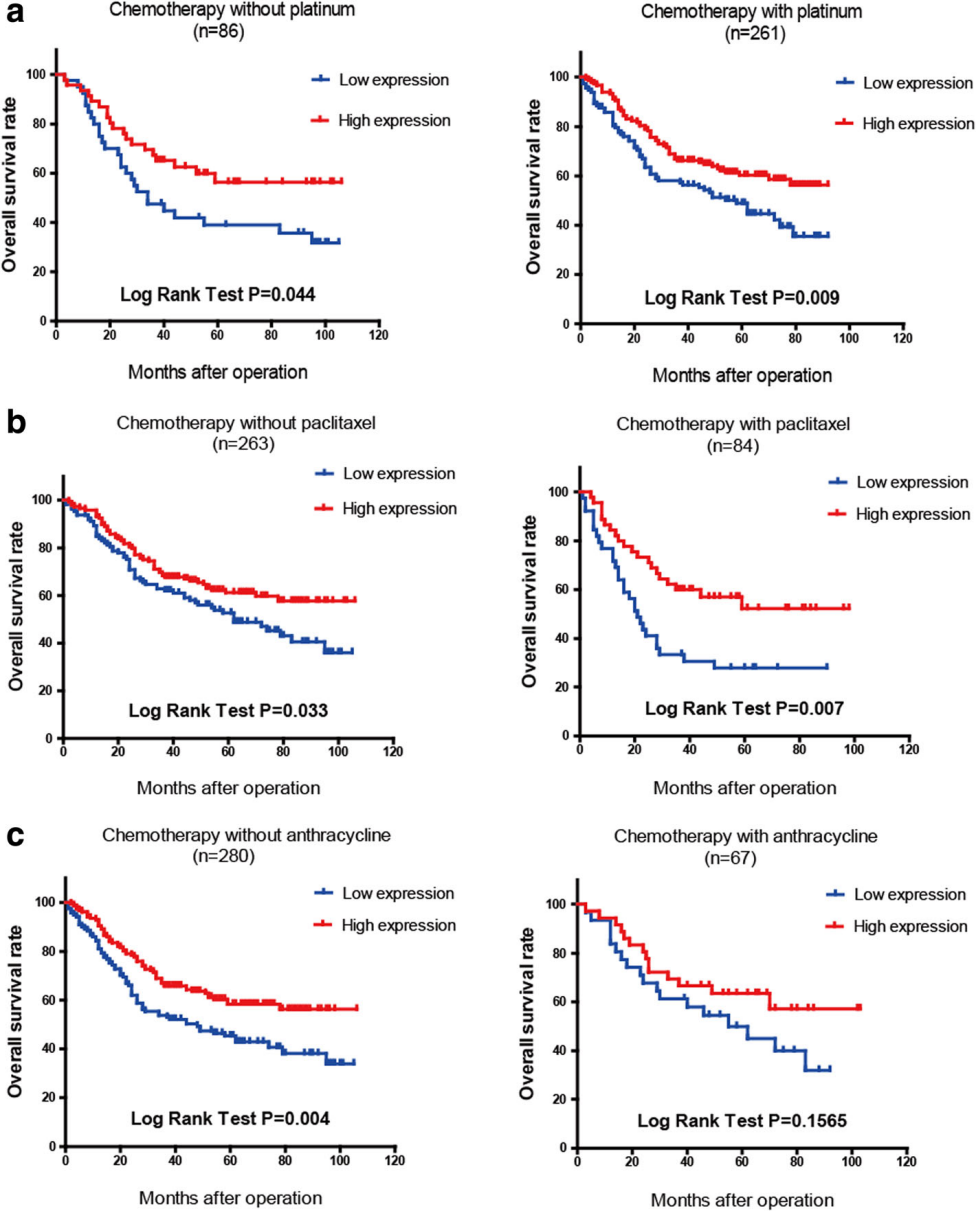
FBXW7 expression correlates with outcome of different chemotherapeutics: **a** Low FBXW7 expression was associated with poor prognosis in patients chemotherapy with or without platinum, especially for 261 patients treated with platinum (*P* = 0.009 vs 0.044). **b** Low FBXW7 expression was associated with poor prognosis in patients chemotherapy with or without paclitaxel, especially for 81 patients treated with paclitaxel (*P* = 0.007 vs 0.033). **c** OS curve of 67 patients treated with anthracycline did not show a stratistical difference between the two groups

Subsequently, 347 patients received postoperative adjuvant chemotherapy. The chemotherapeutic regimens were primary floxuridine-based, with or without platinum, paclitaxel or anthracycline. Among them, 84 patients’ regimens included paclitaxel, 66 patients included anthracycline and 261 patients included platinum. The OS curves showed some significant prognostic correlation between different types of chemotherapeutics and high versus low FBXW7 expression (Fig. 5). The Kaplan-Meier log rank test demonstrated that high FBXW7 patients respond to paclitaxel and platinum better than low FBXW7 patients (*P* = 0.007 and *P* = 0.009 respectively). There was no significant difference between the two groups treated by anthracycline (*P* = 0.1565).

### Univariate and multivariate analyses in advanced gastric adenocarcinoma

Univariate and multivariate Cox proportional hazards model analyses were performed on the clinicopathologic database of the 428 advanced stage patients to evaluate the prognostic parameters associated with overall survival (Table 2). According to the results of the univariate analyses, FBXW7 expression, tumor size, tumor differentiation, T stage, N stage, vessel invasion, nerve invasion and chemotherapy were significantly associated with overall survival. Furthermore, FBXW7 expression, tumor size and N stage were included in a multivariate Cox regression analysis to adjust for the covariates and were confirmed as independent prognostic factors.

**Table 2:** Univariate & multivariate analyses of prognosis parameters for OS in 347 advanced gastric cancer patients

	Univariate analysis			Multivariate analysis		
Prognostic parameter	RR	95% CI	*P* value	RR	95% CI	*P* value
FBXW7 (low vs.high)	0.688	0.527–0.899	0.006*	0.622	0.501–0.875	0.004*
Age (<60 vs. ≥60)	1.441	0.870–1.494	0.340	1.008	0.822–1.440	0.557
Gender (male vs. female)	0.942	0.712–1.246	0.674	0.979	0.734–1.306	0.887
Size (≤5 cm vs. >5 cm)	1.994	1.522–2.612	0.000*	1.539	1.156–2.048	0.003*
Tumor differentiation (well/moderate vs. poor)	1.426	1.406–1.945	0.025*	0.922	0.665–1.280	0.629
T stage (T2 vs. T3/T4)	3.104	2.253–4.277	0.000*	1.504	0.948–2.385	0.083
N stage (absent vs. present)	1.701	1.494–1.936	0.000*	1.404	1.158–1.701	0.001*
Vessel invasion (absent vs. present)	2.115	1.555–2.876	0.000*	1.372	0.975–1.931	0.070
Nerve invasion (absent vs. present)	1.819	1.292–2.561	0.001*	1.183	0.813–1.722	0.380
Chemotherapy (with or without)	0.707	0.509–0.982	0.038*	0.793	0.562–1.119	0.187

*Statistical significant (*P* < 0.05)

## Discussion

In this study, we demonstrated that low level FBXW7 expression is associated with cancer progression and poor prognosis using a large database in gastric cancer. While similar conclusions have been noted in several other types of malignancies, this is the first large cohort study reported in gastric cancer. Moreover, we showed that FBXW7 may serve as a marker for chemotherapeutic sensitivity based on our analysis of corresponding clinical data.

It has been reported that low FBXW7 expression is associated with tumor progression in a diverse array of malignancies including breast, endometrial, ovarian, and pancreatic cancers as well as T-cell acute lymphoblastic leukemia [13, 14, 17]. In our study, gastric cancers with low FBXW7 expression was associated with cancer progression and worse prognosis. FBXW7 is a tumor suppressor that regulates degradation of oncogene proteins such as MYC, cyclin E, Notch, and MCL1 [18]. Yakobori found that FBXW7 mRNA expression level was associated with tumor size, lymphatic metastasis and prognosis in gastric cancer [16]. Our study also found a similar link with prognostic outcome, and further expanded on this by evaluating FBXW7 protein expression. FBXW7 regulates expression of various oncoproteins, but the specific targets and related pathways are not clear in gastric cancer. Yokobori also confirmed that down regulation of Fbxw7 gene expression by RNA interference can lead to c-Myc and cyclin-E accumulation and increased proliferation of gastric cancer cells. C-Myc plays an important role in mitogenesis and cell growth. Cyclin E is a key molecule in cell cycle regulation. Accordingly, both c-Myc and cyclin-E also show disordered expression in gastric cancer tissue [10].

Recently, an association between FBXW7 and chemotherapeutic sensitivity has been noted in several malignancies [19]. Our study shows by IHC data analysis that high FBXW7 expression is linked to an improved response to chemotherapy. However, the anthracycline treatment cohort does not show a similar correlation. Docetaxel, an analogue of paclitaxel, has been widely used in the treatment of advanced and late stage gastric cancer [20, 21]. Wertz found that a pro-survival protein,MCL1, is a crucial regulator of apoptosis triggered by antitubulin chemotherapeutics [22]. The degradation of MCL1 is blocked in patient-derived non-small cell lung cancer tumor cells that either lack FBXW7 or have loss-of-function mutations in FBXW7, in turn conferring resistance to antitubulin agents and promoting chemotherapeutic-induced polyploidy. This may be a mechanism by which docetaxel resistance occurs in gastric cancer. A correlation between cisplatin resistance and FBXW7 expression has also been indirectly reported by Zhou and colleagues [23]. They found MiR-223 promotes cisplatin resistance in human gastric cancer cells via regulation of the cell cycle by targeting FBXW7. Upregulation of FBXW7 partially reverses the effect of miR-223 mimic in 7901/DDP cells, demonstrating FBXW7 plays a crucial role in platinum treatment resistance. Furthermore, Kojiro reported miR-223 regulates FBXW7 leading to trastuzumab-resistance in Her-2 positive GC cells [24]. Overall, these studies suggest that FBXW7 may play an important role in chemotherapy resistance in cancers including gastric cancer.

## Conclusions

In conclusion, we have identified low FBXW7 expression as a potential powerful marker of poor prognosis in patients with gastric adenocarcinoma. Furthermore, in view of the different chemotherapeutics outcomes found in this large database study, FBXW7 may serve as an important predictor in chemotherapeutic responses. However, there are still more studies need to be done to confirm FBXW7’s effectiveness in predicting the responses in specific chemotherapeutic regimen.

## Abbreviations

FBXW7: F-box and WD repeat domain-containing7
GC: Gastric adenocarcinoma
IHC: Immunohistochemistry
MDR: Multidrug resistance
OS: Overall survival
PBS: Phosphate buffer solution
qRT-PCR: Quantitative reverse transcription PCR
SCF: Skp-Cullin1-F-box
